# Plasma rich in growth factors membrane as coadjuvant treatment in the surgery of ocular surface disorders

**DOI:** 10.1097/MD.0000000000010242

**Published:** 2018-04-27

**Authors:** Ronald M. Sanchez-Avila, Jesús Merayo-Lloves, Ana C. Riestra, Silvia Berisa, Carlos Lisa, José Alfonso Sánchez, Francisco Muruzabal, Gorka Orive, Eduardo Anitua

**Affiliations:** a Fundación de Investigación Oftalmológica, Instituto Universitario Fernández-Vega, Oviedo; b University Institute for Regenerative Medicine and Oral Implantology, UIRMI (UPV/EHU-Fundación Eduardo Anitua); c Biotechnology Institute; d Laboratory of Pharmacy and Pharmaceutical Technology, Faculty of Pharmacy, University of the Basque Country; e Networking Biomedical Research Center on Bioengineering, Biomaterials and Nanomedicine, CIBER-BBN, SLFPB-EHU, Vitoria, Spain.

**Keywords:** corneal and ocular surface diseases, fibrin membrane, plasma rich in growth factors, platelet-rich plasma, PRGF, PRP

## Abstract

To evaluate the safety and efficacy of the surgical use of plasma rich in growth factors fibrin membrane (mPRGF) in different ocular surface pathologies.

Fifteen patients with different corneal and conjunctival diseases were included in the study. Patients were grouped according to the use of mPRGF as graft (corneal and/or conjunctival) or dressing; they were also grouped according to the surgical subgroup of intervention (persistent corneal ulcer [PCU], keratoplasty, superficial keratectomy, corneal perforation, and pterygium). Best corrected visual acuity, intraocular pressure (IOP), inflammation control time (ICT), mPRGF AT (PRGF membrane absorption time), and the healing time of the epithelial defect (HTED) were evaluated throughout the clinical follow-up time. Safety assessment was also performed reporting all adverse events.

mPRGF showed a total closure of the defect in 13 of 15 patients (86.7%) and a partial closure in 2 patients (13.3%). The mean follow-up time was 11.1 ± 4.2 (4.8–22.8) months, the mean ICT was 2.5 ± 1.1 (1.0–4.0) months, the mean mPRGF AT was 12.4 ± 2.0 (10.0–16.0) days, and for the global HTED the mean was 2.9 ± 1.2 (1–4.8) months. Results showed an improvement in BCVA in all patients, with an overall improvement of 2.9 in Vision Lines. The BCVA significantly improved (*P* < .05) in the groups of corneal graft and dressing. In the PCU subgroup (6 patients), the healing time of epithelial defect was significantly reduced (*P* < .05) in patients treated only with the mPRGF in comparison to those which mPRGF therapy was associated to the amniotic membrane. The IOP remained stable (*P* > .05) throughout the clinical follow-up time. No adverse events were reported after mPRGF use.

The mPRGF is effective and safe as coadjuvant treatment in surgeries related with ocular surface disorders, being an alternative to the use of amniotic membrane. The mPRGF accelerates tissue regeneration after ocular surface surgery thus minimizing inflammation and fibrosis.

## 1 Introduction

Ocular surface and corneal diseases are usually caused by external agents such as alkali burns, physical or mechanical trauma, or infections. However, they can also be caused by some chronic scarring keratoconjunctivitis mediated in part by autoimmune pathology, including cicatricial ocular pemphigoid, Stevens–Johnson syndrome, atopic keratoconjunctivitis, and peripheral ulcerative keratitis. These pathologies can be complicated by corneal ulcers and perforations that may compromise the integrity of the eyeball and visual viability.^[1]^ In order to heal some corneal defects and reduce the risk of ocular perforation, amniotic membrane transplantation (AMT), tissue adhesives (collagen or fibrin), and tissue patches derived from animals have historically been used, although they are sometimes insufficient.^[2]^ The amniotic membrane (AM) has been used since the early twentieth century. In fact, it was used for the first time in the ophthalmology field in the 1940s in patients who suffered alkali burns to the eyes. The AM stimulates the ocular surface wound healing with minimal inflammation and scarring by combining mechanical protection and biological factors. The main indications for the use of AM include corneal diseases and trauma, and it also includes disorders that involve the conjunctiva, sclera, and eyelids.^[3]^ The AM has been widely used in the management of persistent epithelial defects (PEDs), neurotrophic ulcers, corneal damage caused by chemical agents, pterygium, and corneal surface reconstruction.^[4,5]^ However, there are some limitations in the use of AM, because it is a biological product with great variability in its quality and biological capacity; there is a high potential risk for biological contamination; and its availability is scarce and its cost is high.^[6,7]^

Plasma rich in growth factors (PRGF) is a standardized and optimized technology for tissue repair and regeneration. It is based on the preparation of several autologous formulations obtained from the patient's own blood, including growth factors-rich eye drops, a 3D fibrin scaffold, and a biomimetic and elastic membrane (PRGFs fibrin membrane [mPRGF]).^[8–10]^ The properties of these formulations are similar to those of AM, such as its capability to induce tissue regeneration, its bactericidal and bacteriostatic activity, its anti-inflammatory, and its antifibrotic potential.^[11–14]^ Furthermore, these characteristics have been widely evidenced in multiple preclinical and clinical studies in the ophthalmology field.^[8,10,15,16]^ Particularly, the potential benefits of mPRGF have been evaluated in several studies using it alone or in combination with other membranes like AM or bovine pericardium collagen membrane; in all of these studies, a stable closure of corneal ulcers was observed after treatment with mPRGF with no evidence of infection, inflammation, or pain.^[17–19]^

The mPRGF is able to retain for more than a week almost 30% of the growth factors content involved in tissue regeneration including platelet-derived growth factor-AB, vascular endothelial growth factor, hepatocyte growth factor, and insulin-like growth factor-1, among others.^[13]^ Leukocytes are not collected during mPRGF preparation thus reducing the content in pro-inflammatory proteins (IL-1b, IL-16, among others), characteristic that differentiates the mPRGF from other platelet-rich plasmas (PRPs) membranes.^[12]^ One of the main components of the mPRGF is the fibrin scaffold, in which a 3D fibrin network enriched in growth factors and proteins, interacts with the patient's own extracellular matrix, and cellular components thus promoting tissue regeneration and wound healing.^[11]^

The present work aims to provide preliminary information about the safety and efficacy of the surgical use of mPRGF in different ocular pathologies (corneal and conjunctival) as a graft for corneal and/or conjunctival pathologies or even as a dressing. To the best of our knowledge, this is the first clinical study of translational research that addresses the surgical regeneration of the ocular surface by using autologous mPRGF.

## 2 Materials and methods

### 2.1 Patients

A descriptive and retrospective clinical study was carried out to analyze the results obtained from patients subjected to an ocular surface and cornea surgical pathology using the mPRGF as a single therapy or associated with another surgical technique to achieve an anatomical and functional recovery. The study was conducted at the University Institute Fernandez-Vega (Oviedo, Spain) between June 2015 and May 2017. Informed consent was obtained from all participants included in the study. All the principles of the Declaration of Helsinki were fulfilled to carry out this study. The Institutional Clinical Research Committee approved this study.

The patients included in this study suffered from corneal and/or conjunctival pathologies, and were susceptible to surgical treatment. A total of 15 patients with different surgical pathologies were included: PED, neurotrophic corneal ulcer (NCU), traumatic corneal perforation, failed keratoplasty, band keratopathy, and pterygium with important inflammatory component. These patients did not respond to medical therapies including corticosteroids, nonsteroidal anti-inflammatory drugs, artificial tears, therapeutic contact lenses (TCL), some of them having also failed to AMT therapy and corneal surgeries such as keratoplasty.

The patients were divided in different surgical subgroups: persistent corneal ulcer (PCU, including those types that did not respond to treatment), corneal perforation, keratoplasty, superficial keratectomy, and pterygium removal. The use of mPRGF was grouped according to the surgical application as corneal pathology graft, conjunctival pathology graft, corneal and conjunctival pathology graft, or dressing. Patient's baseline data of gender, age, presurgical diagnosis, time of disease evolution, previous ophthalmic surgeries, and associated findings were obtained from the medical records.

### 2.2 Preparation of the mPRGF

**Figure 1:**
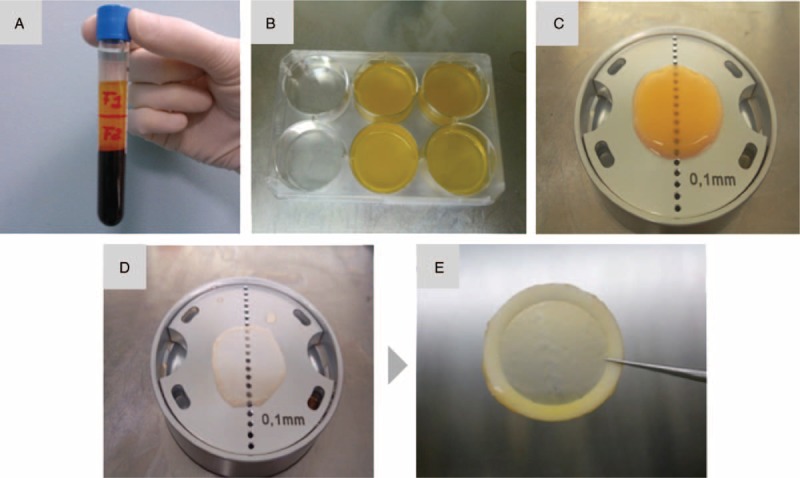
Preparation process of plasma rich in growth factors membranes (mPRGF). (A) Blood tube after centrifugation. (B) Coagulation of fibrin clot. (C) mPRGF on the former. (D) mPRGF obtained after conformation. (E) Membrane mounted on nitrocellulose disk.

To obtain PRGF membranes (mPRGF) for ophthalmic application, 81 mL of blood were collected in 9 mL tubes with 3.8% sodium citrate as anticoagulant. The blood samples were centrifuged at 580g at room temperature for 8 min in an Endoret System centrifuge (BTI Biotechnology Institute, S.L., Miñano, Álava, Spain). The plasma column was collected into fraction 2 (F2) defined as the 2 mL PRP just above the leukocyte buffy coat, and fraction 1 (F1) defined as the remaining plasma volume above the F2. For each membrane, 5 mL of F2 were activated with 10% calcium chloride and incubated in wells of 35 mm diameter at 37°C for 30 min. Once fibrin clots were formed, they were placed in a shaper and pressed for 30 s, obtaining mPRGF of 500 μm of thickness. Then, the mPRGF obtained were mounted on nitrocellulose discs to make their handling easier (Fig. 1).^[20]^ The PRGF eye drops were also manufactured, according to the protocol described by Anitua et al.^[15]^ After the surgery, the PRGF eye drops were applied topically (in the conjunctival sac) 4 times per day for 6 weeks in the affected eye.

### 2.3 Surgical technique

**Figure 2:**
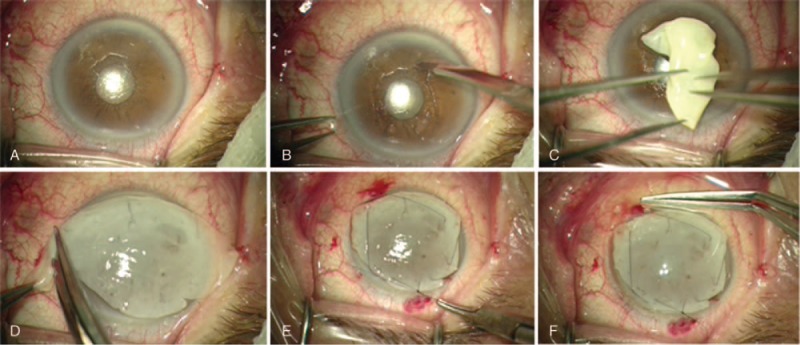
Use of the mPRGF in persistent epithelial defects. (A) Intraoperative image of PED. (B) Epithelium debridement. (C) Placement of mPRGF on corneal surface. (D) Extending and trimming down of mPRGF. (E) Running suture of mPRGF to cornea with 10/0 nylon. (F) Therapeutic contact lens placement over mPRGF after surgery. mPRGF = plasma rich in growth factors membranes, PED = persistent epithelial defect.

**Figure 3:**
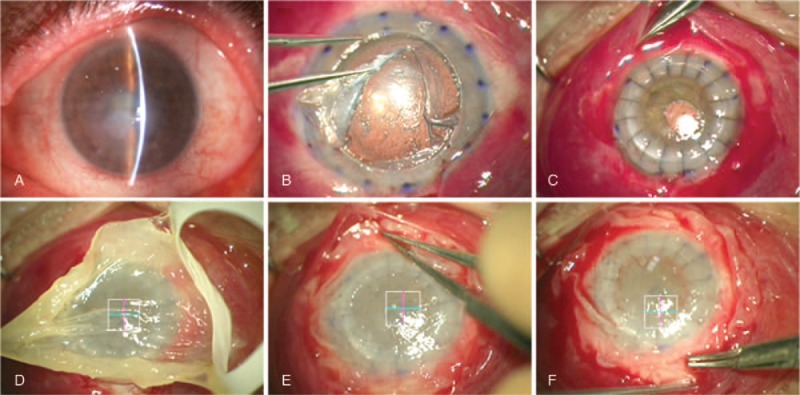
mPRGF treatment after cataract surgery followed by deep anterior lamellar keratoplasty. (A) Leukoma secondary to herpes simplex virus and bacterial corneal abscess. (B) Corneal dissection until predescemet space. (C) Corneal donor button suture with 10/0 nylon and 360° conjunctival peritomy. (D) Application of conformed mPRGF to the ocular surface. (E) Placement of mPRGF inside the subconjunctival space. (F) Running suture of the mPRGF to the conjunctiva with vicryl 8/0. mPRGF = plasma rich in growth factors fibrin membrane.

A retrobulbar anesthesia (mepivacaine hydrochloride, 2%; B. Braun Melsungen AG; Arnhem, Netherlands) was performed in all patients using a maximum of 4 mL of anesthetic. The eyes were rinsed with 20% povidone iodine 15 min after the anesthetic administration. The eyelids were opened with Castroviejo blepharostat. When mPRGF was used as a corneal pathology graft, the injured epithelium was removed mechanically with a surgical knife. Then, mPRGF was placed on the corneal surface, extended and trimmed down to adapt it to the corneal surface. Continuous suture for 360° was carried out with 10-0 nylon to stitch the mPRGF to the sclerocorneal bed, and finally, TCL was placed above the membrane (Fig. 2). When mPRGF was used as a conjunctival pathology graft (pterygium surgery), it was first dissected and cut, the recipient bed was rinse, and then the mPRGF was placed on the surgical bed and trimmed down, nylon 10-0 separated stitches were sutured, and finally, a TCL were placed over the membrane. When mPRGF was used as a dressing, the initial procedure was performed (e.g., cataract surgery by phacoemulsification), followed by corneal transplant surgery such as deep anterior lamellar keratoplasty (DALK), penetrating keratoplasty (PK), or pseudochamber protected keratoplasty (PPK). Subsequently, a 5-mm scleral dissection plane toward the posterior segment was carried out after 360° conjunctival peritomy, then mPRGF was placed, trimmed, and fitted below the dissected conjunctiva, after that a continuous suture with Vycril 8-0 between mPRGF and adjacent conjunctiva was performed. Finally, a TCL was placed on the ocular surface (Fig. 3). Along the postoperative period patients were treated with anti-inflammatory drugs, antibiotic, and TCL. Oral steroids therapy was continued in those patients who were taking chronic treatment.

### 2.4 Postsurgery follow up

All patients were followed daily for 7 days, then once a week for 4 weeks, and finally, each month until clinical resolution. Several outcome variables were collected to measure the efficacy of mPRGF treatment including type of surgery, concomitant surgical treatment, best corrected visual acuity (BCVA in LogMAR), intraocular pressure (IOP, in mm Hg, measured with Icare PRO tonometer, 2015, Finland), inflammation control time (ICT, time at which the relief of the eye is established, no redness, no pain, no ocular surface symptoms), and mPRGF AT (absorption time, time at which the PRGF membrane was completely reabsorbed). Healing was also evaluated under slip-lamp scoring it from 1 to 3; 1—complete healing (ulcer closure), 2—partial healing (small PED < 1 mm), and 3—no healing (large and persistent PED, no ulcer closure); the healing time of the epithelial defect (HTED) was also evaluated, defined as the time in which more than 80% of the epithelial defect has been healed. The clinical follow-up time was recorded for each patient included in the study, as well as the lines of vision gained by each surgery group. The safety of treatment with mPRGF was also evaluated, recording and treating any adverse event or complication that appeared after the application of mPRGF.

### 2.5 Statistical analysis

A descriptive statistic was made using absolute and relative frequency distributions for qualitative variables, and analysis of mean values and standard deviations for quantitative variables. Different normality tests (Kolmogorov–Smirnov and Shapiro–Wilk) were performed on each variable sample. Any potential difference observed between baseline and after mPRGF treatment was analyzed using the statistical paired *t* test. The Mann–Whitney *U* test was used to analyze independent samples. The analysis of variance test was performed for the comparison between groups and the Jonckheere–Terpstra test was also used to evaluate nonparametric variables. The level of statistical significance was set at *P* < .05. The statistical software package SPSS v19.0 for Windows (SPSS Inc., Chicago, IL) was used for all statistical analyses.

## 3 Results

Fifteen patients with corneal and ocular surface pathologies were treated with mPRGF. The mean age of patients was 62.7 ± 17.3 years (ranged from 27 to 82). Three women (20%) and 12 men (80%) were included in the study (Tables 1 and 2). Four cases received oral corticosteroids chronically and their treatment was maintained throughout the follow-up, no ophthalmological complications were observed in these patients. Previously to the mPRGF treatment, 3 patients had received AMT on several times (patients 2, 12, and 13) with poor response to the treatment; 11 (73.3%) patients received therapy with TCL without improvement in the healing of their corneal pathology; and 6 cases (40.0%) had some type of corneal transplantation.

**Table 1:**
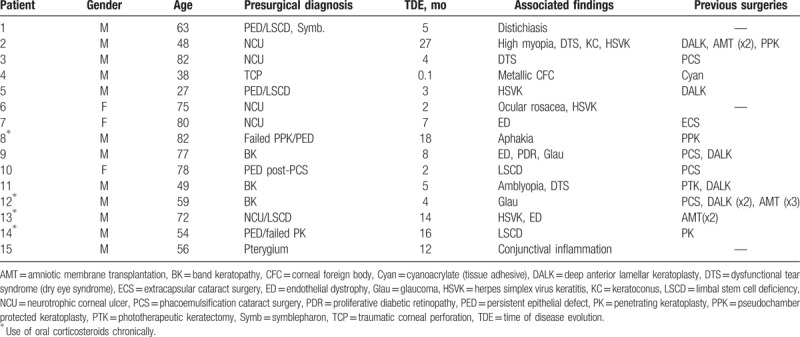
Baseline data of patients.

**Table 2:**
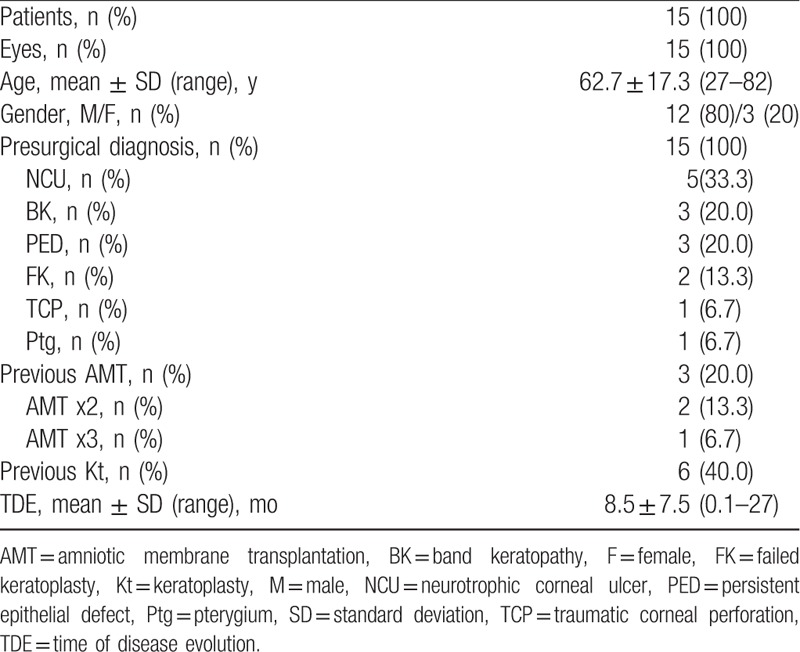
Baseline characteristics of patients grouped by findings.

The frequency of cases according to the surgical subgroup was as follows: PCU included 6 patients (40.0%), keratoplasty included 4 patients (26.7%), 3 patients underwent superficial keratectomy (20.0%), 1 patient underwent corneal perforation (6.7%), and pterygium removal included 1 patient (6.7%). According to the surgical application, mPRGF was used as corneal pathology graft in 7 cases (46.7%), conjunctival pathology graft in 1 case (6, 7%), graft of corneal and conjunctival pathology in 3 cases (20.0%), and dressing in 4 cases (26.7%). The AM was used in 5 patients (33.3%) as a dressing over the mPRGF (3—PCU, 2—superficial keratectomy), and in 4 patients some type of keratoplasty was performed (1 patient underwent PPK, 2 patients underwent PK, DALK + cataract surgery was carried out in 1 patient) was used associated with mPRGF as a dressing. The latter patients received oral prednisone (1 mg/kg, in descending dose for 1 month) within the institution's keratoplasty protocol. All patients were also treated after the surgery with TCL for 1 month (Tables 3 and 4).

**Table 3:**
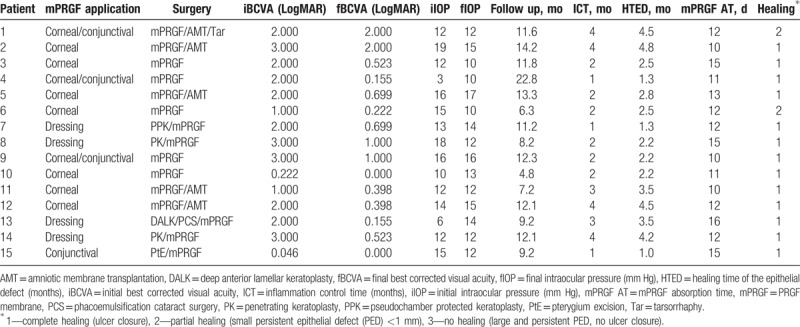
Patient outcomes treated with mPRGF.

**Table 4:**
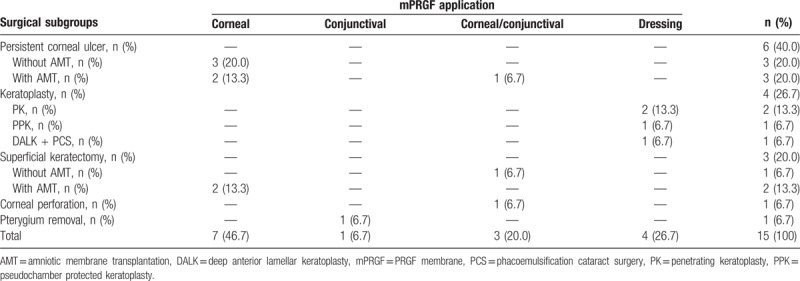
Application of mPRGF according to surgical subgroup.

**Figure 4:**
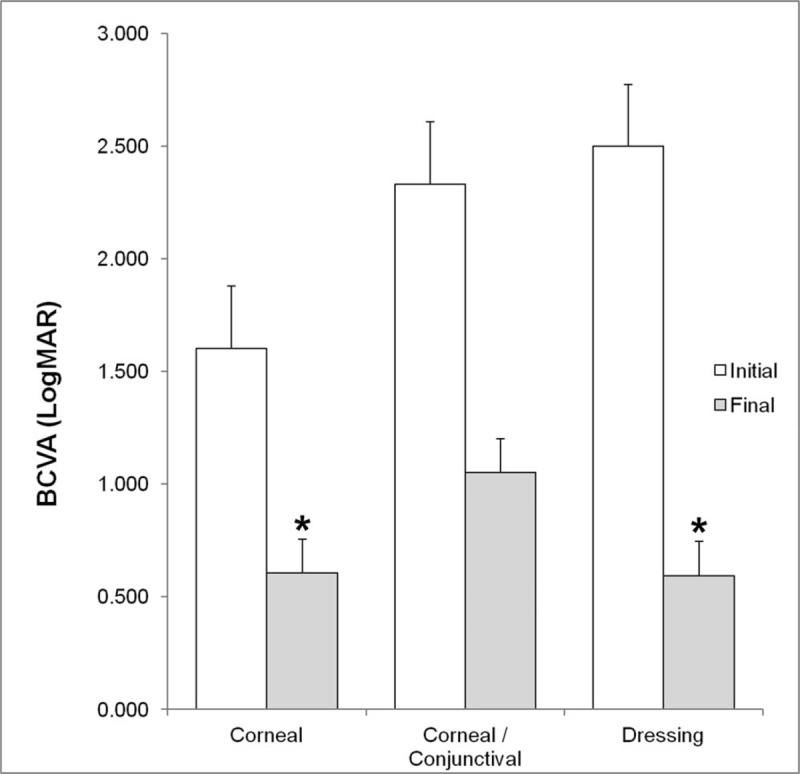
BVCA (LogMAR) outcomes measured before and after mPRGF treatment in the different groups included in the study. ^∗^Statistically significant differences (*P* < .05) before versus after treatment with mPRGF. BCVA = best corrected visual acuity, mPRGF = plasma rich in growth factors fibrin membrane.

The BCVA improved in all patients treated with mPRGF with an overall improvement of 2.9 in Vision Lines. Vision lines improved significantly (*P* < .05) in 3.1 and 3.3 for the corneal graft group and the dressing group, respectively. The improvement was 2.7 for the corneal/conjunctival graft and 1.0 for conjunctival graft (Fig. 4). The baseline overall values of BCVA (LogMAR) were 2.02 ± 0.87 (ranged from 0.22 to 3.00) and after surgical treatment the BCVA was 0.74 ± 0.63 (ranged from 0.00 to 2.00) (*P* = .001), achieving an improvement of the overall visual acuity of 65.4%. An improvement of 47.1% (*P* = .021) in visual acuity was achieved in the PCU subgroup; however, no statistically significant differences were found when BCVA results were compared between subgroups with and without AMT. On the other hand, visual acuity improvement was achieved in the 92% of patients with corneal perforations (Table 5). In the total group, the IOP remained stable throughout the clinical follow-up, with an initial IOP of 12.9 ± 4.2 (3.0–19.0) and a final IOP of 12.9 ± 2.2 (10.0–17.0) mm Hg (*P* = .96). The mean follow-up time was 11.1 ± 4.2 (4.8–22.8) months, the mean ICT was 2.5 ± 1.1 (1.0–4.0) months, the mean mPRGF AT was 12.4 ± 2.0 (10.0–16.0) days, and for the global HTED the mean value was 2.9 ± 1.2 (1–4.8) months. At the final follow-up time, complete healing was observed in 13 patients (86.7%) and partial healing was showed in 2 patients (13.3%). The differences in the time variables for comparisons between surgical subgroups were not statistically significant (*P* > .05).

**Table 5:**
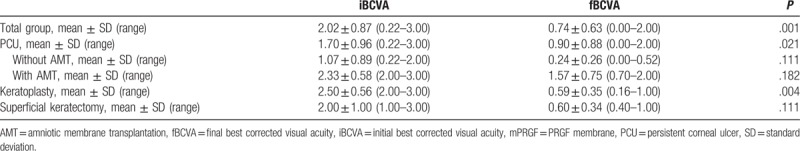
Visual acuity outcomes measured before and after mPRGF therapy.

**Figure 5:**
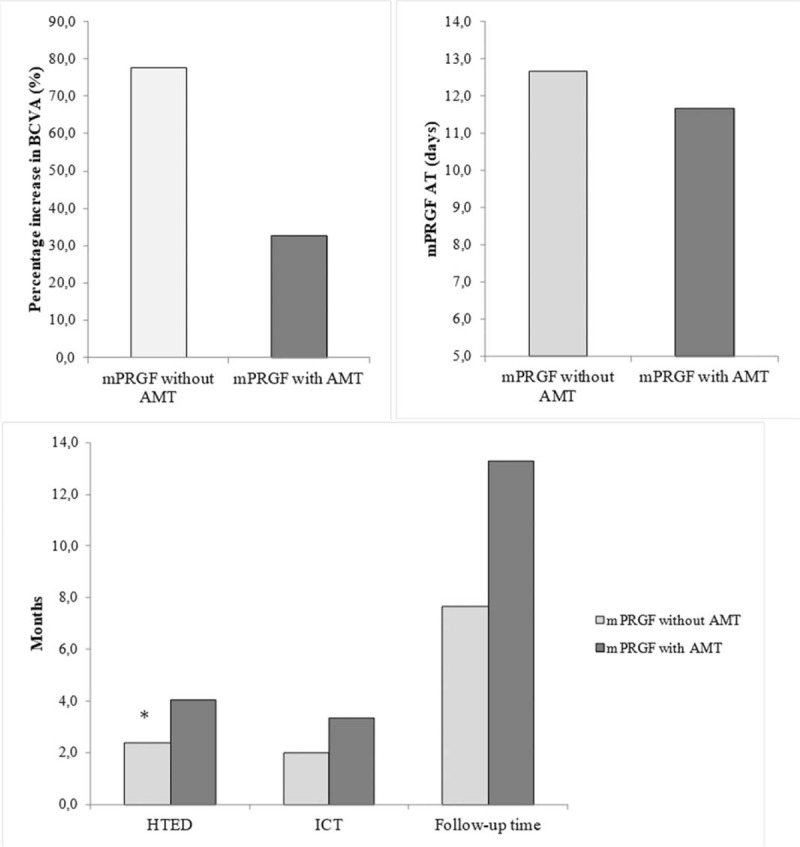
Outcome variables in surgical subgroup PCU according to association with AMT. AMT = amniotic membrane transplantation, BCVA = best corrected visual acuity, HTED = healing time of the epithelial defect, ICT = inflammation control time, mPRGF = plasma rich in growth factors fibrin membrane, mPRGF AT = absorption time of mPRGF, PCU = persistent corneal ulcer. ∗*P* < .05.

When outcome variables were evaluated in the PCU surgical subgroup attending to whether they were treated alone with mPRGF (3 cases) or associated with the AMT (3 cases), the following results were obtained (Fig. 5): The percentage increase in BCVA for the subgroup treated alone with mPRGF was 77.6%, and for the mPRGF group associated with AMT was 32.6% (*P* = .827). HTED variable (months) showed values of 2.4 and 4.0 for the subgroup treated alone with mPRGF and associated with AM, respectively, being these differences statistically significant (*P* = .046). No statistical significances were observed between both subgroups (treated alone with mPRGF or associated with AM) for the rest of the outcome variables measured, being, respectively, of 2.0 and 3.3 for the ICT (months) (*P* = .114); 7.7 and 13.3 months (*P* = .127) for the follow-up time (months); and finally, 12.67 and 11.67 days (*P* = .658) for the mPRGF AT. The variables for the surgical subgroup of superficial keratectomy did not show statistically significant differences among the groups (*P* > .05) depending on whether they had used AMT or not.

**Figure 6:**
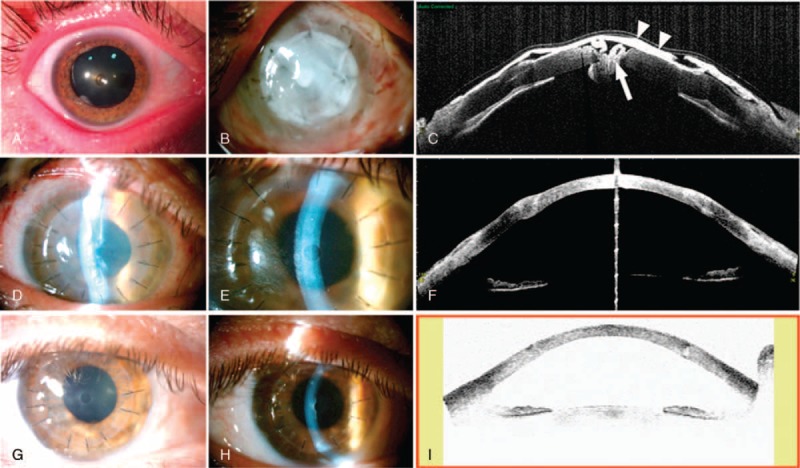
Ocular trauma due to a central corneal metallic foreign body. (A) Corneal foreign body injury with maceration and central thinning. (B) First postoperative day after treatment with a central mPRGF cap and another mPRGF used as a dressing over the first. (C) OCT of anterior segment at first day postsurgery. Both mPRGFs used as a cap (arrow) and as a dressing (arrowheads) are observed. (D–I) Evolution after a second surgery (deep anterior lamellar keratoplasty): (D) Fifth day after surgery (E) Third week after surgery. (F) OCT of anterior segment in third week after surgery. (G) Third month postsurgery. (H) Fifth month after surgery. (I) Visante in the fifth month after surgery. mPRGF = plasma rich in growth factors fibrin membrane, OCT = Optical Coherence Tomography.

Patient number 4 is represented in Fig. 6 showing an ocular trauma due to a central corneal metallic foreign body, which caused maceration and stromal lysis with corneal perforation, and who was urgently surgically treated with mPRGF (a central plug and corneal dressing with a second layer of mPRGF). The latter allowed the corneal stabilization. Then, a corneal transplant (DALK) was performed in a second surgical time. This last patient has a final visual recovery of 7 lines of vision and was stable for 22.8 months of the follow-up period.

No adverse events were reported neither with the use of mPRGF nor with the associated treatments. Patients showed no pain, no discomfort, or any other symptoms during the postoperative follow-up period. No infection or inflammation symptoms were manifested in clinical follow-up time. Only 1 patient required temporary tarsorrhaphy, which was opened 8 days after surgery without any incidents.

## 4 Discussion

The AM has been widely used in surgical pathologies of the ocular surface and cornea, because it promotes epithelialization and exhibits antifibrotic, anti-inflammatory, anti-angiogenic, and anti-microbial properties.^[21]^ However, the AM has some disadvantages such as the need for a tissue bank for its production, the risk of viral infections transmission by the donor, variability in production protocols, and the need for storage.^[22]^ Classically the AM has been used surgically, as a temporary patch or as a permanent graft, however, due to its cellular proliferation properties, it has also been recently used as scaffolding for epithelial limbal stem cell cultures.^[23]^

The ex vivo cultured limbal epithelial transplantation with AM as surgical therapy for the treatment of limbal stem cell deficiency has been shown to be effective in recovering the ocular surface and improving visual acuity.^[24]^ It has been observed in in vitro studies that the AM can maintain the limbal epithelial stem cells quiescent, which supports the utility of the AM in the ex vivo cultured limbal epithelial transplantation.^[25]^ In another in vitro study, it was demonstrated the superior ability of carbodiimide cross-linked denuded amniotic membrane against the denuded AM to preserve limbo-corneal epithelial progenitor cells, finding that could be crucial for future basic investigations using the AM as scaffold.^[26]^

Platelets play an important role in homeostasis, preventing blood loss at sites of vascular injury. At the same time they constitute a natural reservoir of growth factors, adhesive proteins, and cytokines within their alpha granules.^[27]^ These proteins and other molecules stored in the alpha-granules are involved in tissue repair and healing processes. Autologous PRP preparations have been used since 1999 to accelerate tissue regeneration process in different medical fields such as maxillofacial surgery, tendon repair, joint surgery, ulcer treatment, and dental surgery.^[28–31]^

PRPs include a large list of techniques and therapies generally distinguished by concentrating platelets and sometimes leukocytes within a plasma liquid volume. Most of these approaches allow the formation of fibrin scaffolds for the differentiation of several cell types thanks to the physical and mechanical properties.^[32]^ However, the wide range of PRP protocols reported so far show significantly different clinical results leading to general confusion. Anitua et al demonstrated that leukocyte-free fibrin scaffolds obtained with PRGF (PRGF-Endoret) technology were more stable than those containing leukocytes. Furthermore, leukocytes inclusion modifies the content in growth factors increasing the concentration of proinflammatory cytokines.^[12]^

Optimal alternatives to cell carrier materials for ocular surface reconstruction should be optically transparent, biocompatible and should have enough biomechanical strength to be able to suture it to the ocular surface tissues and it should be reproducible in their production at low cost.^[2]^

Interestingly, other authors have found similar results to the ones presented herein. For example, Alio et al described the use of autologous fibrin membrane as a safe and effective alternative for the treatment of corneal ulcers healing.^[19]^ The autologous origin of fibrin membrane is a clear advantage with respect to other biomaterials such as AM or bovine pericardium, especially the mPRGF due to the possibility of preparing into the hospital and even in the same operating room, unlike the previous ones that depends on the availability of the tissue centers.^[33]^ The effect of platelet-rich fibrin (PRF) membrane has been investigated in an animal model with conjunctival lesion. This study demonstrated the beneficial effect for the conjunctival healing, due to its chemical and support effects for cell growth and proliferation; the autologous PRF membrane is a growth factors-enriched endogenous scaffold very useful for ocular surface reconstruction.^[34]^

AM applications have been previously described for the treatment of PEDs, neurotrophic ulcers, chemical damage, pterygium, and conjunctival surface reconstruction with clear benefits.^[4]^ However, this treatment shows some drawbacks such as the risk to suffer a biological contamination, the high variability of clinical results due to the nonstandardized production protocols, the low availability of this product, and because it is an expensive treatment.

mPRGF has been used in several medical fields including dermatology, dentistry, traumatology, and orthopedics for the treatment of different medical situations such as chronic cutaneous ulcers, Achilles tendon surgery, and oral implantology.^[29,35,36]^ To our knowledge, this is the first translational research study that successfully uses mPRGF for treating corneal and ocular surface pathologies in a cohort of patients with different indications such as PED, band keratopathy, NCU, corneal perforation, failed keratoplasty, and pterygium.

Several studies have demonstrated that PRGF technology has similar biological features as AM, for instance regenerative, anti-inflammatory, antifibrotic, and anti-microbial properties.^[37–39]^ However, it is important to highlight some advantages of mPRGF when compared with AM and other biomaterials. Some of these include the standardized protocol of PRGF, which will ensure the reproducibility of the treatment, the less economic impact related to its preparation and use, the fast clinical availability, and the advantage in terms of its autologous origin, avoiding the risk of viral or prions transmission. Furthermore, it is also important to highlight that PRGF eye drops are obtained in the same process of mPRGF preparation. PRGF eye drops can be used in the postsurgical treatment, thus accelerating the ocular surface tissue regeneration.

The present study presents some limitations that should be considered. One important issue is that the study has a retrospective design. Furthermore, the number of patients included in the study is low and their initial diagnosis is variable. However, this study has an adequate follow-up time with good functional and structural results. Future randomized clinical studies with a higher number of patients will be necessary to properly confirm these initial results.

Our preliminary results suggest that mPRGF which is easy to prepare from the patient's own blood and it would be available for its use in the operating room in 40 to 60 min, could be an alternative if AM would not be available. Results show a complete healing in the 87% of patients included in the study and a significant improvement of BCVA in all patients treated with mPRGF with no adverse effects reported. Furthermore, it is important to highlight that mPRGF and PRGF eye drops could be obtained in the same process, using the latter as postsurgical treatment for the ocular surface regeneration in the postoperative period. In addition, mPRGF can be applied in those pathologies in which corneal and conjunctival epithelium are compromised and in several types of corneal transplants as regenerative treatment thus minimizing inflammation and fibrosis processes.

## Acknowledgment

The authors would like to thank Virginia Cuadrado for her support with the English grammar.

## Author contributions

**Conceptualization:** Jesus Merayo-Lloves, Jose Alfonso, Eduardo Anitua.

**Data curation:** Ronald Mauricio Sanchez-Avila, Silvia Berisa, Carlos Lisa.

**Formal analysis:** Ronald Mauricio Sanchez-Avila, Francisco Muruzabal.

**Funding acquisition:** Jesus Merayo-Lloves, Jose Alfonso, Gorka Orive, Eduardo Anitua.

**Investigation:** Ronald Mauricio Sanchez-Avila, Ana Cristina Riestra, Silvia Berisa, Carlos Lisa, Jose Alfonso, Francisco Muruzabal.

**Methodology:** Ronald Mauricio Sanchez-Avila, Ana Cristina Riestra, Silvia Berisa, Carlos Lisa, Jose Alfonso, Francisco Muruzabal.

**Project administration:** Jesus Merayo-Lloves, Jose Alfonso, Gorka Orive, Eduardo Anitua.

**Resources:** Jesus Merayo-Lloves, Francisco Muruzabal, Gorka Orive, Eduardo Anitua.

**Software:** Ronald Mauricio Sanchez-Avila, Francisco Muruzabal.

**Supervision:** Ana Cristina Riestra, Jose Alfonso, Gorka Orive, Eduardo Anitua.

**Validation:** Jesus Merayo-Lloves, Carlos Lisa, Francisco Muruzabal.

**Visualization:** Carlos Lisa.

**Writing – original draft:** Ronald Mauricio Sanchez-Avila, Carlos Lisa, Jose Alfonso, Francisco Muruzabal.

**Writing – review & editing:** Ronald Mauricio Sanchez-Avila, Ana Cristina Riestra, Silvia Berisa, Carlos Lisa, Jose Alfonso, Francisco Muruzabal, Gorka Orive, Eduardo Anitua.
